# Overcoming the Bitter Taste of Oils Enriched in Fatty Acids to Obtain Their Effects on the Heart in Health and Disease

**DOI:** 10.3390/nu11051179

**Published:** 2019-05-27

**Authors:** Aleksandra Stamenkovic, Riya Ganguly, Michel Aliani, Amir Ravandi, Grant N. Pierce

**Affiliations:** 1 Institute of Cardiovascular Sciences, St Boniface Hospital, Winnipeg, MB R2H2A6, Canada; astamenkovic@sbrc.ca (A.S.); gangulr@gmail.com (R.G.); aravandi@sbgh.mb.ca (A.R.); 2 Department of Physiology and Pathophysiology, Rady Faculty of Health Sciences, University of Manitoba, Winnipeg, MB R3E0W3, Canada; 3 Canadian Centre for Agri-Food Research in Health and Medicine (CCARM), Albrechtsen Research Centre, St Boniface Hospital, University of Manitoba, Winnipeg, MB R2H2A6, Canada; maliani@sbrc.ca; 4 Department of Human Nutritional Sciences, Faculty of Agricultural and Food Sciences, University of Manitoba, Winnipeg, MB R2H2A6, Canada; 5 Internal Medicine, Rady Faculty of Health Sciences, University of Manitoba, Winnipeg, MB R3E0W3, Canada

**Keywords:** bitter taste, fatty acids, heart disease, metabolism, atherosclerosis, ischemia

## Abstract

Fatty acids come in a variety of structures and, because of this, create a variety of functions for these lipids. Some fatty acids have a role to play in energy metabolism, some help in lipid storage, cell structure, the physical state of the lipid, and even in food stability. Fatty acid metabolism plays a particularly important role in meeting the energy demands of the heart. It is the primary source of myocardial energy in control conditions. Its role changes dramatically in disease states in the heart, but the pathologic role these fatty acids play depends upon the type of cardiovascular disease and the type of fatty acid. However, no matter how good a food is for one’s health, its taste will ultimately become a deciding factor in its influence on human health. No food will provide health benefits if it is not ingested. This review discusses the taste characteristics of culinary oils that contain fatty acids and how these fatty acids affect the performance of the heart during healthy and diseased conditions. The contrasting contributions that different fatty acid molecules have in either promoting cardiac pathologies or protecting the heart from cardiovascular disease is also highlighted in this article.

## 1. The Challenge of Incorporating Fatty Acid into the Diet

Dietary intake of fatty acids is important for human and animal health. Indeed, this is particularly clear with essential fatty acids which are not found in the body and must be ingested to be present. Essential fatty acids like alpha-linolenic acid (ALA), an omega-3 fatty acid, and linoleic acid (LA), an omega-6 fatty acid, are necessary for human health. However, despite the requirement for these fatty acids in human health, the acceptability of oils that contain fatty acids in the diet is a significant challenge due to their taste characteristics. This can be compounded by limited shelf stability of oils that may compromise taste and aroma further through unwanted oxidation of the fatty acids found in the oil. 

Analytical methods used for studying the compounds that cause bitterness in food include chromatography and mass spectrometry, but also sensory evaluation. The so-called “electronic tongue” is a simple device used to analyze the compounds in food using chemical sensors in a similar way as the human tongue analyses taste. Busch et al. validated the efficacy of the electronic tongue in detecting bitterness in virgin olive oils by conducting a study in which they compared the enzyme-based biosensors and a standard HPLC method for the analyses of the total content of phenolics in virgin olive oils [1].

Olive oil and flaxseed oil (also known as linseed oil) are two commonly used culinary oils. Both oils exhibit a bitter and pungent taste [2,3,4,5,6]. This can limit their acceptance in the general population and restrict their use in both regular diets and in experimental research studies as well [7]. Different agricultural practices, new processing technologies, and educational campaigns to emphasize the benefits of an association of a bitter taste with health have all been suggested as strategies to improve the acceptance of these oils in the general population [3]. However, the acceptance of the bitterness in oil is influenced by the country of origin of the consumer. Spanish consumers were more tolerant of the pungent and bitter taste characteristics of olive oil than were their American counterparts [6]. The bitter taste originates from the unique polar polyphenol-rich composition of the oils [3]. Extended storage of flaxseed oil can result in its deterioration and generation of methionine oxidation of its cyclolinopeptides [2]. The primary one responsible for the bitter taste of flaxseed oil has been identified as cyclic octapeptide cyclo (PLFIM OLVF) [5], also referred to as cyclolinopeptide E [2].

The bitter taste can be reduced in a variety of ways. From a food processing standpoint, both high and low temperatures can be used as effective ways of reducing the bitter taste. Storage of olive oil at 5 °C for two to eight weeks leads to a reduction in bitterness [8]. In addition, Garcia et al. showed that heating the olives leads to a decrease in olive oil bitterness and the reduction in bitterness correlated with the time and temperature used for heating [9]. Heating the olives did not cause any changes in the oxidative stability of the olive oil or its acidity, however, Garcia et al. did find decreased levels of phenolic content. Not only heat, but also cold temperatures can be effective in reducing the bitterness of olive oil. Another method used successfully to reduce the bitterness of olive oil is the addition of phospholipids. Koprivnjak et al. [10] showed that the addition of 5–10 g of soy lecithin/kg olive oil leads to a significant reduction in bitterness and an increase in the sweetness of olive oil. The partitioning of polyphenols between oil and water phases can be an effective method used to decrease the total polyphenol content and therefore leads to a reduction in bitterness without affecting the physicochemical parameters of oil [11]. Adding spices [4] or flavorings to the oils can also change the taste characteristics in a way to try to make the oil more compatible. Shape Foods, Inc. in Brandon. Manitoba Canada produced a variety of flavored flax oils in dessert flavors (chocolate, caramel, banana, and coconut) and culinary flavors (Italian, Mexican, Indian, Thai, Szechuan and Culinary).

Fish, hempseed, flaxseed, and olive oil can be ingested as an oil by the spoonful, in salad dressings, by pill form or incorporated into food products [7,12,13,14]. The oils provide the fatty acids to the body with greater bioavailability than foods that contain the same amount of fatty acids [13]. However, each of these types of oils (i.e., fish versus hemp versus flax) has a different bioavailability to the body [14]. Fish oil had its eicosapentaenoic acid (EPA)/docosahexanoeic acid (DHA) content absorbed better than the ALA in flaxseed oil, which was more bioavailable than the LA in hempseed oil [14]. However, although the bioavailability and the biological benefit of each of these fatty acids differ considerably, encouraging the consuming public to actually ingest these oils has been a considerable challenge. The incorporation of these fatty acids into foods commonly consumed disguises their bitter taste characteristics and encourages greater acceptability [7,12]. For example, even though bioavailability may be compromised slightly [13], the inclusion of milled flaxseed into biscuits, muffins, bagels, bread products, and even pasta can increase consumption [13]. In 2011, Aliani and colleagues used a trained sensory panel to test whether adding different flavors will reduce the bitterness of muffins and snack bars containing milled flaxseed [7]. Flaxseed is rich in the omega-3 fatty acid ALA. Among several flavors used such as apple spice, gingerbread raisin, orange cranberry, and cappuccino chocolate, the gingerbread raisin flavor reduced the bitterness of flax best. Therefore, the authors suggested that the flavorings that naturally contain a small amount of bitterness could be effective in reducing the bitterness caused by flax [7].

The food processing industry is beginning to recognize this potential and is starting to incorporate healthy fatty acids into foods without compromising taste. Ultimately, this will introduce these beneficial fatty acids into the daily diet of the general public to allow them to access their heart-health benefits.

## 2. Introduction to the Biological Activity of Fatty Acids 

Fatty acids play a role in energy generation and storage, phospholipid membrane formation, and signaling pathways. Therefore, fatty acid metabolism can be catabolic with respect to energy and metabolite generation or anabolic for creating biologically important molecules from dietary sources of fatty acids.

Fatty acid metabolism plays a particularly important role in the heart. The continuous pumping action of the heart exhibits a high energy demand. ATP is the ultimate source of this energy. In order to generate ATP at a high enough rate to sustain cardiac contractility, almost all ATP (−95%) is derived from mitochondrial oxidative phosphorylation [15]. The remainder is derived from glycolysis within the tricarboxylic acid (TCA) cycle. Beta oxidation within the heart is under complex control. The factors which affect the cycle include the supply of fatty acid, the presence of competing substrates, the energy demand of the heart and the supply of oxygen to the heart [15]. Fatty acids can be recognized by the heart as free fatty acids (FFA), triacylglycerol (TAG), or very low-density lipoproteins (VLDL). In the case of VLDL, the enzyme lipoprotein lipase (LPL) can hydrolyze TAG and VLDL within cardiomyocytes. Regulation of LPL plays a significant role in fatty acid metabolism within the heart. Increased LPL translocation to the plasma membrane has been correlated with increased beta oxidation [16]. Mitochondrial uptake of fatty acids is regulated by carnitine palmitoyltransferase (CPT1). The enzyme CPT1 catalyzes and converts long-chain acyl-CoA into long-chain acyl-carnitine. This facilitates uptake into the mitochondria and will have distinct effects on rates of beta oxidation. Changes in fatty acid metabolism within the heart have been implicated in a variety of complex cardiovascular diseases.

Once fatty acids are taken up by the cell through transport proteins and/or passive diffusion, they can then be converted into energy. This process is known as beta oxidation. Once the fatty acid is taken up within the cell, a CoA group is added to the fatty acid using the enzyme fatty acid CoA synthase (FACS). This forms long-chain acyl-CoA. Carnitine palmitoyltransferase 1 (CPT1) converts long-chain acyl-CoA to long-chain acylcarnitine. This allows the fatty acid moiety to be transported into the inner mitochondrial membrane via carnitine translocase. The inner membrane protein CPT2 then converts long-chain acylcarnitine back to long-chain acyl-CoA inside the mitochondria. The long-chain acyl-CoA then enters the beta oxidation pathway. Fatty acid beta oxidation consists of breaking down the long-chain acyl-CoA molecule into acetyl-CoA. Four main enzymes are used in the process to convert acyl-CoA to acetyl-CoA. The number of acetyl-CoA molecules is dependent upon the carbon chain length in the fatty acid. Acetyl-CoA can then enter the citric acid cycle and electron transport chain to yield ATP [15].

Other than beta oxidation, fatty acids can also be used in other key processes within the cell. Of note, polyunsaturated fatty acids (PUFAs) are integrated into phospholipids within the cell membrane. Structurally, phospholipids (or glycerophospholipids) consist of a glycerol backbone, two fatty acids and a phosphate containing a polar head group. PUFAs are preferentially incorporated into the sn-2 position of phospholipids. Incorporation of omega-3 PUFAs into membrane phospholipids can lead to changes in membrane properties and alter the function of membrane proteins [17,18]. It has been shown that the antiarrhythmic effect of omega-3 PUFAs is achieved by their incorporation into membranes and modulation of ion channel function in cardiomyocytes [19]. 

On demand, specific PUFAs can be cleaved from the phospholipid by phospholipase A_2_ (PLA_2_) [20]. Once cleaved, PUFAs can undergo enzymatic oxygenation and are transformed into short term lipid mediators known as oxylipins [20]. Moreover, PUFAs on the sn-2 position of phospholipids can be oxidized leading to the production of oxidized phospholipids which were recently shown to be involved in the formation and progression of various cardiovascular pathologies [20,21,22,23,24,25,26].

Oxylipins represent a class of fatty acid metabolites present in all tissues in the body and produced by oxygenation of polyunsaturated fatty acids by three different enzymes. The tissue oxylipin profile depends on many different factors. Since oxylipins are derived from polyunsaturated fatty acids, the amount of dietary PUFAs influences oxylipin production. Moreover, the presence of different enzymes, as well as enzyme preferences for certain PUFAs, will influence the tissue oxylipin profile. The major, but not the only source of oxylipins, are fatty acids released from membrane phospholipids by the cytosolic PLA_2_ [27]. Upon releasing fatty acids from the phospholipids, these fatty acids are converted to oxylipins by three main pathways: cyclooxygenase (COX), lipoxygenase (LOX) and cytochrome P-450 (CYP) epoxygenase and omega-hydrolase [28].

## 3. Dietary Fatty Acids and Cardiovascular Disease

**Figure A1:**
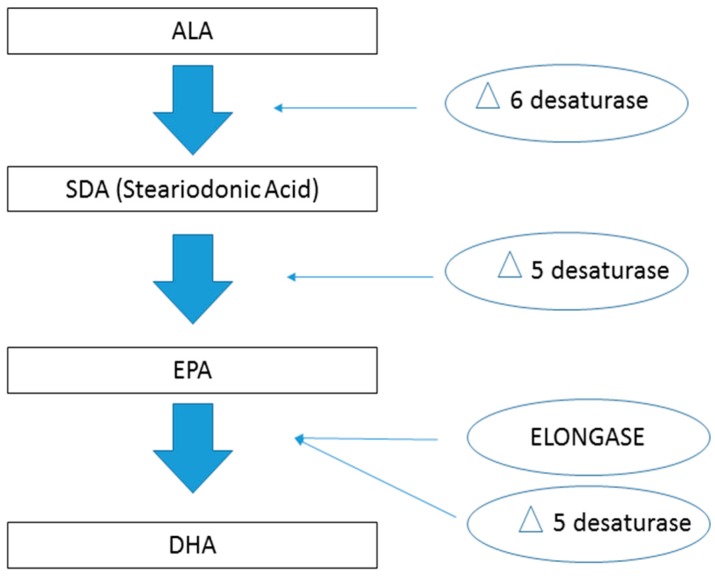
Metabolism of ALA into EPA and DHA by specific enzymes. ALA: alpha linolenic acid; EPA: eicosapentaenoic acid; DHA: docosahexanoeic acid.

There has been a great deal of interest in two distinct categories of PUFAs on cardiovascular disease, the *n*-6 and *n*-3 fatty acids [29]. The position of the double bond, either in the 3rd or 6th position from the methyl end group will distinguish an *n*-6 fatty acid from an *n*-3 fatty acid. Within the cell, these ‘essential’ fatty acids are metabolized and converted into physiologically recognized compounds [30]. Long-chain *n*-3 fatty acids like ALA can be converted into two longer chain derivatives (DHA-docosahexaenoic acid and EPA-eicosatetraenoic acid), primarily by the 5- and 6-desaturase enzymes (Figure A1). DHA is derived from EPA by *β*-oxidation. *n*-6 fatty acids such as linoleic acid (LA) are converted into AA using the 5- and 6-desaturases similar to *n*-3 fatty acids. A higher ratio of *n*-6: *n*-3 intake in the diet will shift the desaturase action to favor the production of AA [30].

Essential fatty acids can only be found in dietary sources and are required for adequate function of the human body. Essential fatty acids cannot be made by the human body. It is important to note that there are two major sources of essential fatty acids: alpha-linolenic acid (ALA) and linoleic acid (LA). ALA is an *n*-3 PUFA found in a variety of food items such as walnuts and flaxseeds. However, it is most prevalent in flaxseed. LA is an *n*-6 PUFA enriched in safflower, poppyseed and rapeseed oil [31]. These two fatty acids serve in a variety of cell maintenance functions. There is evidence, however, suggesting that over-consumption of *n*-6 PUFAs may lead to increased inflammation within the body. It is important to note that fatty acids (essential or nonessential) can be found in a variety of dietary sources. Depending upon the saturation, different fatty acids are found in different foods. PUFAs are generally found in fish, flaxseed and grape seed oils. Saturated fatty acids (SFAs) are found in palm, coconut and cottonseed oils as well as chocolate products [32]. Further research into some of these fatty acids to better elucidate their effects within the body and more specifically, their mechanism of action, will aid in the ongoing research within the field of nutraceuticals and functional foods [33,34,35,36,37].

Unlike *n*-6 fatty acids, *n*-9 fatty acids are primarily found in foods such as olive oil, rapeseed oil, mustard seeds/oil, and wallflower seeds. Omega-9 fatty acids are usually found in two forms, oleic acid (18:1 *n*-9) and erucic acid (22:1 *n*-9). Both of these fatty acids have important biological functions. For example, oleic acid, found in olive oil, has been shown to decrease inflammation [32]. Erucic acid is enriched in colewort or kale. Some of the early studies suggested no severe effects of erucic acid on the heart [38], however, more recent evidence has correlated high intake of erucic acid with complications within the heart [39]. Therefore, erucic acid intake should be limited to 2% of total energy intake [40].

Dietary fat recommendations for cardiovascular health have focused on limiting the intake of saturated fatty acids. Studies of the association between saturated fatty acid intake and cardiovascular disease has generated conflicting data, with some dietary guidelines recommending to limit SFA intake and other studies showing no connection between SFA and CVD. A meta-analysis of 21 cohort studies showed no association between SFA consumption and an increased risk of CVD and coronary heart disease (CHD) [41]. However, the key to cardiovascular health may be the type of nutrient that replaces SFAs. An analysis of 11 cohort studies showed that the replacement of 5% energy intake of SFAs with PUFAs was associated with decreased risk of coronary events and mortality [42]. Another two prospective cohort studies reported a 25% and 15% reduction in CVD risk when SFAs are replaced with PUFAs and monounsaturated fatty acids (MUFAs), respectively [43]. Conversely, the replacement of SFAs with carbohydrates augmented the risk of coronary events [42].

Omega-3 fatty acids are found in a variety of sources. Marine oils contain two major *n*-3 fatty acids. EPA and DHA have important anti-inflammatory and early developmental functions within the body [44]. Unlike ALA, DHA and EPA can be derived within the body through different enzymes known as desaturases. ALA is an essential fatty acid and can be a precursor for DHA and EPA. However, ALA is not completely converted to both EPA and DHA. ALA is a plant-derived *n*-3 PUFA and can be found in walnuts, sunflower seed, and flaxseed. ALA has important biological actions within the heart [35,45]. In the National Heart, Lung and Blood Institute (NHLBI) study, it was demonstrated that LA and ALA decrease the risk of heart disease by 40% and 70%, respectively [46].

Trans fatty acids (TFAs) are unsaturated fatty acids in which the double bond is in the trans configuration. Artificial TFAs are produced during industrial hydrogenation of vegetable oils leading to solidification and an increase in the half-life for the purpose of prolonged storage in the food industry. Natural TFAs are produced in the rumen of ruminants as a product of bacterial transformation and are found in small amounts in dairy and meat products [47]. The most common industrial TFA is elaidic acid (18:1, omega-9), while vaccenic acid (18:1, omega-7) represents the major ruminant TFA [48]. According to the current guidelines of the European Food Safety Authority, TFA intake should be <1% of the total caloric intake. North Americans consume between 5–10 g/day of industrial TFAs daily which constitutes approximately 2–5% of total energy within our diets [34]. However, some studies suggest that some individuals consume up to 20 g/day of industrial TFAs [49,50]. A 2% increase in TFA consumption leads to a 23% increase in the risk of CHD [51]. TFAs have been associated with an increased risk of CVD by altering serum lipid composition. They increase the ratio of total cholesterol to high-density lipoprotein cholesterol which is an important predictor of CVD [52]. In addition, they increase the levels of low-density lipoprotein (LDL) cholesterol and reduce the levels of high-density lipoprotein cholesterol [52], triglyceride levels [53], and increase Lp(a) levels [54]. Increased dietary TFA intake induces inflammation in women as confirmed by increased levels of interleukin-6 and C- reactive protein [55]. Therefore, the effect on CVD can be indirect, since inflammation is an independent risk factor for atherosclerosis [56]. Furthermore, there is evidence that increased TFA intake leads to endothelial dysfunction by increasing the levels of intercellular adhesion molecule 1, soluble vascular cell adhesion molecule 1, and E-selectin [57]. Apart from playing a role in the development of atherosclerosis, TFA can have an influence on atherosclerotic plaque stabilization. The type of atheromatous plaque with a thin <65 µm fibrous cap which is infiltrated by macrophages [58] is most prone to rupture [59]. In a cross-sectional study of the association between TFAs and a thin fibrous cap atheroma as assessed by optical computed tomography, an association was identified between serum levels of elaidic acid and the presence of a thin fibrous cap atheroma in patients with CHD [60].

In addition to simply avoiding the ingestion of TFAs, nutritional interventions can be an effective way of altering the deleterious effects of trans fatty acids on atherosclerosis development. In a mouse model of atherosclerosis, intake of ground flaxseed, or an ALA-rich flaxseed oil prevented atherosclerotic plaque development induced by mild or moderate TFA intake [35].

## 4. Polyunsaturated Fatty Acids in Atherosclerosis

Atherosclerosis is a chronic disease characterized by lipid deposition and inflammation and hardening of the arteries. An atherosclerotic plaque can be asymptomatic or, as it progresses, it can become unstable, or it can rupture leading to myocardial infarction. Fatty acids are involved in plaque formation and stabilization. Dietary fatty acids can affect atherosclerosis in both a negative or a positive way depending on their structure. In a study conducted in 111 patients with CAD, fatty acid profiles established by gas chromatography showed significant correlation between trans fatty acids: elaidic acid (C18:1trans 9), trans isomers of linoleic acid (trans C18:2 isomers) and trans 11 eicosanoic acid (C20:1 trans 11) and biomarkers of oxidative stress, inflammatory parameters (hs-CRP, IL-6, TNF–α) as well as vascular severity [38]. Increased trans fatty acid intake correlates with an increase in LDL, leading to a promotion of atherosclerosis [61]. An association between saturated fatty acid intake and CVD has been extensively studied. The first trial known as the “Seven Countries Study” had more than 12.000 participants within the seven countries: United States, Finland, Italy, Yugoslavia, Greece, Japan, and the Netherlands. It showed that saturated fatty acids are the most powerful lifestyle predictor of CVD [62]. However, the most recent study that enrolled >135,000 patients, aged 35–70 years in the 18 countries, with over seven years follow-up showed that increased SFA intake was not associated with major CVD outcomes [63].

Omega-6 and omega-3 fatty acids play an important role in cardiovascular disease and health. Conversion of omega-6 fatty acids like LA to arachidonic acid and the conversion of omega-3 fatty acids to DHA and EPA is a process driven by the same enzymes. Therefore, the conversion of ALA will depend on the amount of ALA and LA ingested, but not their ratio [64]. While LA is easily converted to arachidonic acid, ALA conversion to EPA and DHA is 1% and 5% respectively [65].

Taking this into consideration as well as the omega-6 rich and omega-3 poor western diet [66], there is a risk of cardiovascular disease in western societies [67]. The omega-6 derived arachidonic acid (AA) serves as a precursor for the formation of oxylipins via cyclooxygenase (COX) [68]. Oxylipins have both beneficial and deleterious effects on the cardiovascular system. The best-characterized oxylipins are the ones derived from AA, and so far, the levels of AA-derived oxylipins in plasma have shown detrimental effects on cardiovascular health. Oxylipins derived from arachidonic acid promote inflammation [69,70], oxidative stress [71], and vasoconstriction [71,72]. The very first product of COX-induced oxidation of AA is prostaglandin G2 (PGG2) and subsequently, prostaglandin H2 (PGH2) which is further converted to other PGs and thromboxanes through PG and thromboxane synthase [73]. Prostaglandin E2 (PGE2) is involved in cardiac hypertrophy and ischemia [74,75]. Thromboxane A_2_ has potent pro-aggregatory and vasoconstrictive properties [76,77,78]. Its concentration is reduced by aspirin administration [76,77,78]. 20-hydroxyeicosatetraenoic acid (20-HETE) induces vasoconstriction of porcine coronary arteries [79], whereas, 16-, 18-, 19- and 20-HETE metabolites promote vasodilation [80,81]. LOX-derived oxylipins affect the heart by promoting inflammation. Leukotrienes (LTs) lead to endothelial dysfunction, foam cell formation, and smooth muscle cell proliferation, all of which lead to atherosclerosis [82].

Not all oxylipins have a detrimental effect on the heart. CYP enzymes are highly expressed in the heart, and they are involved in converting AA to eicosanoids, which have a cardio-protective role [83,84,85]. Some of them, such as epoxy-eicosatrienoic acid (EpETrE), attenuate ischemia/reperfusion injury [84]. Moreover, EpETrE improved the electrical activity of the heart following ischemia/reperfusion injury [86,87]. This protective role is induced through multiple pathways involving mitochondrial and sarcolemmal potassium channels, p42/p44 MAPK (mitogen-activated protein kinase), and PI3K-AKT [88].

Dietary modulation of fatty acids can influence plasma oxylipin levels. Therefore, dietary fatty acid content has to be carefully considered since the oxylipins derived by specific dietary PUFAs can have a detrimental effect and lead to cardiovascular disease. For example, dietary LA leads to an increased production of 13-hydroxy-octadecadienoic acid (13-HODE) which has been shown to be increased in oxidized LDL, leading to its accumulation in macrophages, and ultimately to atherosclerosis [89].

In contrast, the metabolism of dietary omega-3 fatty acids can be protective in atherosclerosis, since it results in the production of oxylipins that can compete with the ones derived from omega-6 fatty acids and lead to a decreased cardiovascular risk [90,91,92]. Dietary supplementation with omega-3 fatty acids EPA and DHA show protective effects in the heart [31,93]. Epoxyeicosanoids derived by oxidation of EPA/DHA by CYP enzymes, such as 17,18-epoxy-EPA and 19,20-epoxy-DHA, shows potent antiarrhythmic effects [94]. The Singapore Chinese Health study included 744 acute myocardial infarction cases and 744 matched controls and showed not only an inverse association between acute myocardial infarctions and long-chain omega-3 PUFAs and stearic acid but also a positive correlation between AA and the risk of an acute myocardial infarction [95].

The fatty acid composition of chylomicrons can influence lipid deposition in macrophages that leads to atherosclerosis [96,97]. The chylomicrons that are rich in saturated fatty acids are more readily taken up by macrophages compared to the ones that contain omega-3 fatty acids [98]. Omega-3 fatty acids may also protect against atherosclerosis through a reduction in inflammatory signaling by decreasing the expression of various cytokines, as well as by a reduction in adhesion molecules [99]. EPA and DHA also promote the stabilization of atherosclerotic plaques, reducing the occurrence of acute fatal and nonfatal cardiovascular events [100]. Dietary flaxseed supplementation, through its rich content of ALA, inhibits the progression of atherosclerosis and promotes the regression of atherosclerotic plaque in animal models [93].

## 5. Fatty Acids and Ischemic Heart Disease

Fatty acid metabolism is altered in patients post myocardial infarction. In the healthy heart, 95% of the energy is produced by oxidative phosphorylation in mitochondria with the rest coming from glycolysis [101]. During myocardial ischemia/reperfusion injury, metabolism in the heart is significantly altered. During ischemia, the oxygen supply/demand ratio increases leading to a diminished mitochondrial oxygen phosphorylation in proportion to the decreased blood flow [101]. Under these conditions, glycolysis becomes the primary generator of ATP while glucose oxidation is inhibited [101]. The decoupling of these two processes results in the accumulation of lactate and H^+^ [102]. Restoration of blood flow, however, leads to increased fatty acid β oxidation due to fatty acid accumulation, further diminishing glucose oxidation and leading to cellular acidosis [103]. This suggests that the inhibition of fatty acid oxidation can be beneficial during reperfusion. The only clinically available drug that can suppress fatty acid oxidation is trimetazidine. It inhibits 3-ketoacyl CoA thiolase [104], leading to decreased fatty acid oxidation, improved contractility, decreased injury and increased cardiac efficiency [104].

Phospholipids play an important role in ischemic reperfusion injury in the heart. Due to the presence of double bond PUFAs on the sn-2 position, phospholipids are highly susceptible to oxidation. This ultimately leads to the production of oxidized phospholipid species [105,106,107]. Using mass spectrometric analyses, a significant increase in oxidized phosphatidylcholine (oxPC) species was found following in vitro ischemia/reperfusion injury [107]. These oxidized phosphatidylcholine species have been predominantly fragmented species identified as 1-palmitoyl-2-(5′-oxo-valeroyl)-*sn*-glycero-3-phosphocholine (POVPC), 1-palmitoyl-2-(9′-oxo-nonanoyl)-*sn*-glycero-3-phosphocholine (PONPC), 1-palmitoyl-2-azelaoyl-*sn*-glycero-3-phosphocholine (PAzPC) and 1-palmitoyl-2-glutaryl-*sn*-glycero-3-phosphocholine (PGPC) [107]. The exogenous addition of these compounds to cardiomyocytes resulted in cell death, therefore, an increase in these oxPC species during reperfusion injury can be of great clinical importance [107].

Apart from a pharmacological approach for improving heart function and reducing the extent of ischemia/reperfusion injury, there is evidence that dietary intervention might be beneficial as well. A case-control study including 125 patients showed that ALA intake leads to a decreased risk of fatal ischemic heart disease [75]. The Lyon Diet Heart Study, a randomized secondary prevention trial demonstrated that the Mediterranean diet enriched in ALA leads to a 70% reduction in total deaths and 75% reduction in nonfatal myocardial infarctions [108]. In vitro treatment of adult rat cardiomyocytes with ALA led to multiple beneficial effects. It induced increased incorporation of ALA into membrane phospholipids, led to a reduction in cardiomyocyte cell death during ischemia/reperfusion injury [105]. Moreover, ALA treatment led to a decrease in production of oxidized phosphatidylcholines: 1-palmitoyl-2-(5′-oxo-valeroyl)-*sn*-glycero-3-phosphocholine (POVPC) and 1-palmitoyl-2-glutaroyl-*sn*-glycero-3-phosphocholine (PGPC) [105].

## 6. Fatty Acid Metabolism in the Heart in Obesity

Although there is a clear association between fat intake and obesity [109], it is important to recognize that the type of fat ingested may influence obesity risk. This is particularly important with respect to *n*-3 fatty acids. For example, *n*-3 fatty acids may have a protective effect on cardiovascular diseases. However, epidemiological studies remain unclear whether *n*-3 PUFA intake decreases weight gain and abdominal fat. Although some studies have indicated a decrease in body fat with up to 6 g/day EPA and DHA consumption [110], other large prospective health studies such as the Nurses’ Health Study observed an increased prevalence of obesity with high fish intake and *n*-3 PUFA intake [111]. Taken together, these results cannot define a clear role of *n*-3 fatty acids in the prevalence of obesity, however, published data tens to indicate a decrease in obesity risk with *n*-3 fatty acid consumption. As well, most studies are limited by solely analyzing the effect of the *n*-3 fatty acids EPA and DHA on weight gain.

The effects of dietary *n*-6 PUFAs may be less controversial. Rat and mice work suggests that maternal intake of LA predisposes the fetus to AA, the metabolic bi-product of LA. Increased exposure of the fetus to AA has been associated with increased adipocyte production suggesting increased maternal LA intake may predispose an individual to increased adipocyte production [112,113]. Furthermore, studies conducted by Massiera and colleagues [66] suggest that when rat diets were supplemented with 55% LA as a % of the total lipid fraction (19% total energy) for four generations, there was a progressive weight gain in each of four generations [66]. This evidence suggests that the early development of obesity may be correlated to increased *n*-6 fatty acid consumption. Taken together, these studies suggest that *n*-3 and *n*-6 PUFAs have differential effects on obesity. However, more study is necessary to fully understand this phenomenon.

A diseased condition can either cause changes in fatty acid uptake or its oxidation, or, conversely, the disease can be caused by a dysfunction of the fatty acid uptake/oxidation processes. Understanding the role of proteins and pathways involved in fatty acid uptake and the role that changes in metabolism may play is particularly important in diseases of the heart. Since substrate utilization changes during stressful situations, analyzing fatty acid uptake and oxidation patterns are critical to a better understanding of the progression of cardiac diseases.

For example, insulin resistance in the heart and skeletal muscle is associated with a reduced response to increased circulating free fatty acids [114]. This is a common occurrence in insulin-resistant conditions such as obesity and type 2 diabetes [114]. When the uptake of fatty acids exceeds the rate of beta-oxidation, intramuscular lipids can accumulate leading to lipotoxicity [15]. CD36, therefore, plays an important role in increasing long-chain fatty acid (LCFA) uptake into the heart and increasing lipid accumulation when beta oxidation rates do not meet the energy needs of the heart [15]. Treatment with an inhibitor of CD36, sulfo-*N*-succimidyl-palmitate (SSO), decreased LCFA uptake and the subsequent accumulation of lipids in the heart. CD36 also plays a role in atherosclerosis since CD36 is a class B scavenger protein and associated with macrophage infiltration. CD36 inhibition also leads to complications in the heart, such as development of hypertrophy [115]. Taken together, these studies suggest a clear role of CD36 in the progression of CVD. However, while CD36 knock-out may play a beneficial role in limiting disease progression (such as type 2 diabetes), it is still important for basal metabolic function. Long term use of agents such as SSO that inhibit FA uptake through the blocking of CD36 increase the risk of cardiac hypertrophy in rats [116]. Conversely, type 2 diabetes is associated with increased intramyocardial levels of TG [117] which, despite the early association with ventricular dysfunction, can have beneficial effects. Increased intramyocardial TG as well as elevated levels of serum PUFAs were protective in the failing heart [118] and can be important for maintaining ventricular function in both healthy individuals [119] as well as individuals with type 2 diabetes [120].

## 7. Conclusions

Fatty acids represent a class of molecules with a large structural and functional diversity. They have a detrimental role in the heart functioning as a primary energy source in controlled conditions. The main point of this review was to highlight the metabolism of fatty acids in the heart in health and disease as well as to draw the attention to the importance of dietary fats. Dietary fatty acid intake can have both detrimental as well as beneficial effects on heart fatty acid metabolism. However, due to their bitter taste, fatty acid ingestion could be reduced. Here, we summarized the studies describing different methods that could be used to reduce the bitterness of fatty acids in order to improve their ingestion and the beneficial effects these ingested fatty acids, particularly PUFAs may have on our cardiovascular health.
